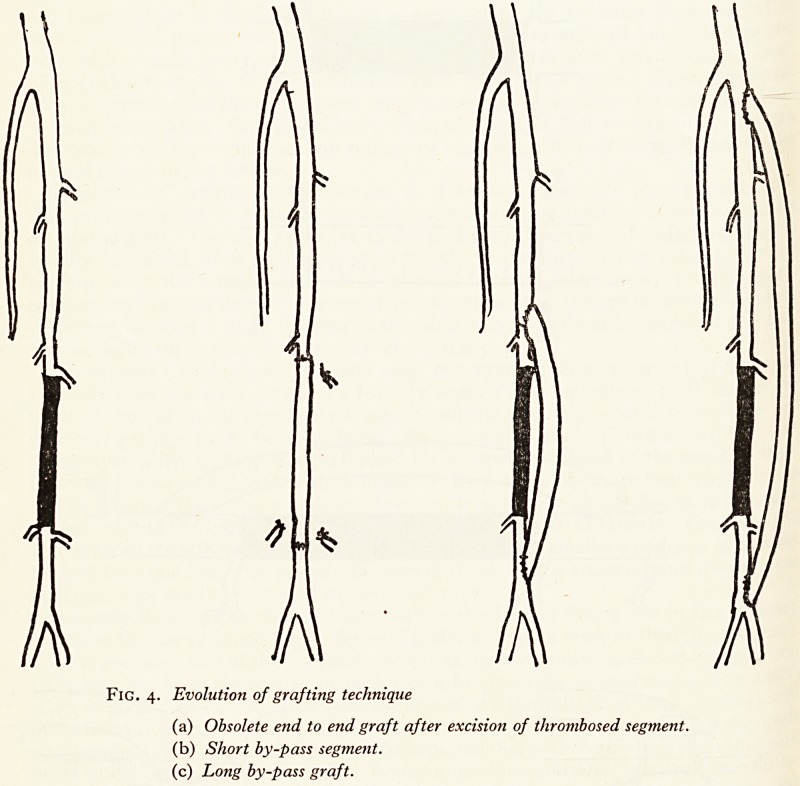# Modern Developments in Peripheral Vascular Surgery

**Published:** 1958-10

**Authors:** R. E. Horton

**Affiliations:** Consultant Surgeon, United Bristol Hospitals


### SOME MODERN DEVELOPMENTS IN PERIPHERAL VASCULAR SURGERY

BY

R. E. HORTON, M.B.E., M.S., F.R.C.S.
*Consultant Surgeon, United Bristol Hospitals*

One of the most important advances in arterial surgery has been the advent of direct operations upon arteries including the use of various forms of grafting, and these have partly displaced operations on the sympathetic nervous system. The largest field for this work is the ever increasing problem of atherosclerosis but no less important, though less common, is the place of direct surgery in patients with injuries involving arteries.

Modern arterial surgery requires an artery bank, and some form of artery bank has been in existence in Bristol since 1952. In a few cases the material that has been used for grafts has been autogenous vein or a plastic prosthesis, but homologous artery has proved to be by far the most suitable material and, until recently, the segments have been taken in the post-mortem room under sterile conditions and stored until needed. Full details of this artery bank and a new method for sterilizing artery segments is to be the subject of a separate communication elsewhere; a summary of the advantages and disadvantages of the various materials used in grafting are given in Table I.

TABLE I

The advantages and disadvantages of various materials used in grafting.

| | Advantages | Disadvantages | Indication |
|---|---|---|---|
| Homograft | Ease of handling. Good long term results. | 1. Need for bank. 2. Supply difficulty. 3. Growth does not take place. | Any form of arterial replacement. |
| Heterograft | Availability. | High incidence of rupture. | NONE. |
| Venous autograft | Availability. Cells of graft survive. | Dilatation when not supported. Only short length available. | Grafting in thigh where muscle or fascial support available. Short lengths e.g. Trauma. |
| Prosthesis | Availability. | High incidence of thrombosis in arteries under 6 mm diameter | Replacement proximal to inguinal ligament. |

ARTERIAL TRAUMA

War provides much experience in the surgical management of trauma, and the first serious attempts to restore damaged arteries were made in World War II. Although sporadic attempts to treat arterial injuries by suture had been made since the time of Carrel's classic work fifty years ago, the standard treatment for many years was

PLATE I

*False aneurysm of the profunda femoris artery associated with a fracture of the femur.*

ligature of the artery proximal to the site of injury. Ligation of the common femoral, or popliteal artery causes a critical degree of ischaemia of the leg. Injury to the superficial femoral artery and its subsequent ligation is attended by a less dangerous reduction in blood flow to the foot but the subsequent interference with function may still be considerable. In World War II De Bakey and Simeone (1945) recorded an amputation rate of 50 per cent in 2,471 arterial injuries treated at first by ligation. In 81 cases treated by suture the rate was reduced to 36 per cent. The Korean War afforded the opportunity to study this problem further and in fact Hughes (1955) was able to report an amputation rate as low as 11 per cent in cases of arterial injury treated by suture.

In peace time, arterial trauma is usually less extensive than that seen in war, and is thus more readily amenable to repair. If lateral or end-to-end suture is impossible with modern advances it is usually possible to implant an arterial graft. In most traumatic cases no serious technical difficulty exists because patients are usually healthy and the artery wall is substantially normal and holds sutures well.

Lateral injury to a main artery results in massive pulsating haematoma. Such a lesion is best approached through the haematoma after controlling the artery above and is repaired with interrupted sutures. In a recent case operated on by this technique the haematoma contained 5½ pints of blood and clot (Plate I).

Arteriovenous fistula is a late result of injury to artery and vein. Quadruple ligation and excision was the standard treatment up to ten years ago and because of the great hypertrophy of the vascular system in this condition serious ischaemia rarely resulted. However, with modern technique it is usually possible to preserve continuity of both artery and vein either by lateral suture of the fistulous opening in the artery or by end-to-end suture after excision (Fig. 1).

Restoration of the circulation is always an urgent matter whether the obstruction is due to embolus or trauma. In either case the urgency is dictated by the adequacy of the collateral vessels. When these are insufficient an important early sign is loss of cutaneous sensation. If serious ischaemia persists for more than 8–10 hours, muscle death will occur and the leg will be lost even if the circulation is eventually fully restored. In cases of embolus heparin may be used to prevent propagation of thrombosis but surgery must not be withheld if sensation is impaired. Heparin must never be used when there is an arterial injury. We have not found sympathetic block or vasodilators of any value in these cases. The only factor which improves the circulation is gravity and when the blood supply is precarious the limb should be placed a little below heart level.

### EFFECTS OF ATHEROSCLEROSIS

With an ageing population the problem of arterial narrowing due to atherosclerosis with consequent claudication or gangrene is becoming more important.

The blood flow through the narrowed artery is progressively impeded until thrombosis takes place and this extends proximally and distally from the site of obstruction to the level of branches which are wide enough to carry a good flow of blood (Fig. 2). The final length of thrombosed artery always has collateral arteries arising flush with the upper and lower limits of the thrombus.

An artery already narrowed by atheroma may thrombose while the patient is confined to bed with an illness or after an operation. In other cases thrombosis happens at night and the patient notices his first symptoms on rising; but in the majority of cases the symptoms increase gradually and no special time of onset is noted.

When a major artery is obstructed by an embolus there is an abrupt change from a normal circulation to total arrest and symptoms are usually dramatic and severe. In atheroma the artery becomes progressively narrowed and the final episode of thrombosis does not give rise to such acute symptoms. Sometimes the thrombosis adds so little to the obstruction which is already present that it is clinically silent and the

Case 1

Case 2

FIG. 1. *Two examples of arteriovenous fistula. In case 1 the artery was repaired by end to end suture after excision of the fistula. In case 2 it was possible to do a lateral repair of the artery. The bomb fragment which caused this fistula is seen in the wall of the artery. The associated false aneurysm of artery or vein seen in both cases is a common finding.*

patient cannot give an accurate date on which it happened, but when thrombosis occurs in an artery which has only suffered a partial occlusion the effect may be considerable.

Atheromatous plaques may be generalized throughout the arterial system but in many cases they are localized to a restricted part of the circulation. A single block with good collaterals may not cause severe symptoms but a second block or severe narrowing gives rise to a much more serious state of ischaemia.

The general picture in atheroma is one of gradual circulatory deterioration. However, each incident of thrombosis is associated with some dilatation of collaterals and the natural tendency is towards some temporary improvement in many cases. In addition, subjective relief and apparent improvement results when the patient with claudication finds that he can walk considerably further if his speed is reduced. These natural phenomena must be taken into account when assessing the value of any new form of treatment.

*Different Diagnosis.* The only serious difficulty appears to be the distinction from sciatica or hip disease. In patients with aortic thrombosis walking may induce ischaemic pain in the buttocks; this gluteal claudication may be mis-diagnosed as pain due to osteoarthritis of the hip. Palpation of the femoral pulse clears up this difficulty immediately. On the other hand patients who have sciatica may be mis-diagnosed as intermittent claudication. This mistake will be avoided if the peripheral pulses are felt because if they are present the patient is not suffering from vascular obstruction and it is necessary to look elsewhere for an explanation of pain in the limb.

*Arteriography*. In practice the only special investigation used to confirm diagnosis is arteriography, and this is only done when direct surgery is considered a possibility. Femoral arteriography is performed by percutaneous puncture; 30 ml. of 50 per cent diodrast is injected and a series of X-ray pictures exposed down the leg. Lumbar aortography is performed by a similar technique. No serious complications have so far been encountered in Bristol, though rare misadventures are well recognized. In aortography, injection of a high concentration of diodrast into the renal or mesenteric circulations may have severe consequences. When one or both femoral pulses are absent the visceral branches of the aorta are certain to receive a particularly heavy dose of diodrast and for this reason the concentration used must not exceed 50 per cent in any circumstances.

a b c

FIG. 2. (a) *Narrowing of the femoral artery by atheroma.*
(b) *Secondary thrombosis.*
(c) *Extension of thrombosis to the origin of the profunda femoris artery.*

*Mis-Use of Vasodilator Drugs.* Many patients attending the Out-patient Department for consultation about peripheral vascular problems are already taking vasodilator drugs. Though subjective remission is often reported there seems no objective evidence that any benefit results. In fact the use of a vasodilator drug by the oral route causes a generalized vasodilation and some lowering of blood pressure results. An additional strain is placed on the heart which is likely to be affected by atheroma and heart failure or fibrillation results in some cases. At the same time the fall in systemic blood pressure is likely to be associated with a reduced blood flow to the limb with a main artery obstruction. Blood flow studies show this to be the case. This criticism applies to all vasodilators used by the oral route. When the intra-arterial route is used the drug passes rapidly through the capillary bed and the effect s again generalized. On the evidence it appears certain that vasodilator drugs can do nothing but harm and that any subjective improvement must be attributed to the natural course of the disease.

*Treatment of Arterial Obstruction due to Atheroma.* The only form of treatment that provides relief in intermittent claudication is the restoration of circulation by an arterial graft. The only exception to this is that the operation of endarterectomy is usually successful when applied to short lengths of the aorta or iliac arteries. When performed on the femoral artery it is followed by early thrombosis. Lumbar sympathectomy does not improve the blood flow to the muscles though in cases presenting with early necrosis a slight increase in skin flow may sometimes be achieved and this may be sufficient to save the leg or to relieve rest pain.

*Intermittent Claudication.* In many cases this sympton is a nuisance but does not seriously interfere with a patient's life. In such a case an arterial graft cannot be justified, but when the incapacity is severe and the patient is unable to work or get to work, grafting must be considered. In a previous study (Horton 1956) it was shown that when grafting was performed for obstructions distal to the inguinal ligament there was an immediate thrombosis of the graft in about 50 per cent of cases and in addition there was a high incidence of delayed thrombosis due to progressive disease beyond the limits of the graft during the ensuing 6–12 months. Grafts to the iliac arteries were more satisfactory and showed a much smaller incidence of thrombosis in the same follow up period. Because of these results, grafts beyond the inguinal ligament were rarely performed in 1956 and 1957.

Recently there has been a tendency both in the United States and in this country to advise the use of much longer femoral grafts to bypass most of the femoral artery and the proximal part of the popliteal artery. At the same time the end-to-end grafting technique has been abandoned in favour of a by-pass type of operation. This operation is greatly to be preferred because immediate thrombosis is most unusual and in addition it is not necessary to sacrifice any collateral arteries in putting the graft into position. (Fig. 3). It is therefore to be expected that with the by-pass technique and using grafts up to 14 in. long, much better immediate and long term results will be obtained. Femoral grafts are therefore being performed again more readily for intermittent claudication and the early results are very encouraging (Fig. 4).

*Gangrene.* When gangrene is advanced the arterial disease is usually severe and grafting is impossible. In a few cases a graft may permit a minor amputation but most patients require an amputation above the knee. The patient with minor necrosis of a toe associated with severe rest pain may also require a major amputation. However, some satisfactory results of grafting are seen in this type of case and if it is technically possible, a graft should be advised because restoration of the circulation will save the leg.

Not all patients in clinical need of treatment by grafting are anatomically suitable. This can only be determined by arteriography which must show that beyond the block there is a patent artery of sufficient size to take the graft. The distal third of the popliteal artery is often suitable for attachment of a graft when the more proximal artery is very diseased. In addition there must be sufficient arterial bed in the leg to a b

FIG. 3. (a) *This end-to-end operation is now obsolete. Note sacrifice of collaterals.*
(b) *Short by-pass operation. Collaterals are not ligated.*

take an adequate flow; at least one of the three named arteries below the knee must be freely patent.

FIG. 4. *Evolution of grafting technique*

(a) *Obsolete end to end graft after excision of thrombosed segment.*
(b) *Short by-pass segment.*
(c) *Long by-pass graft.*

### SUMMARY

The management of cases of arterial obstruction requires individual appraisement of every case. Those clinically requiring surgical intervention are investigated by arteriography and, if careful selection is made on clinical and radiographic evidence, very good results may be expected.

### REFERENCES

De Bakey, M. E. and Simeone, F. A. *Ann. Surg.* (1945), **127**, 534.
Horton, R. E. *Brit. Med. J.* (1956). **1**, 81.
Hughes, C. W. *Ann. Surg.* (1955). **141**, 297.

## Figures and Tables

**Figure f1:**
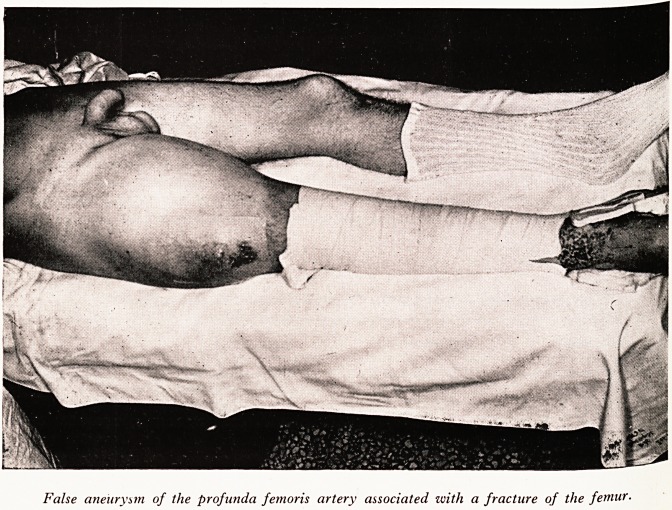


**Fig. 1. f2:**
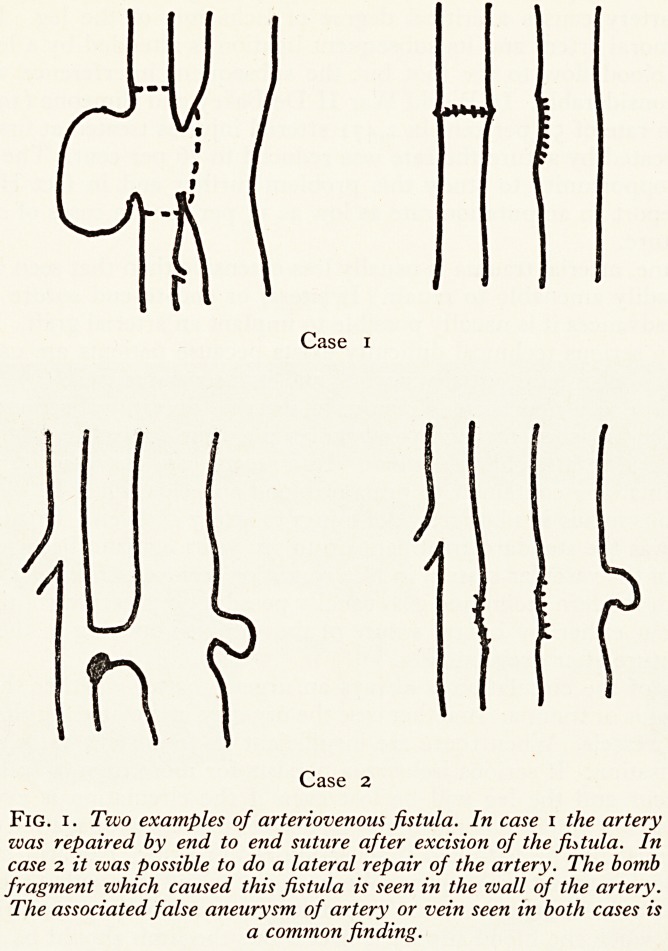


**Fig. 2. f3:**
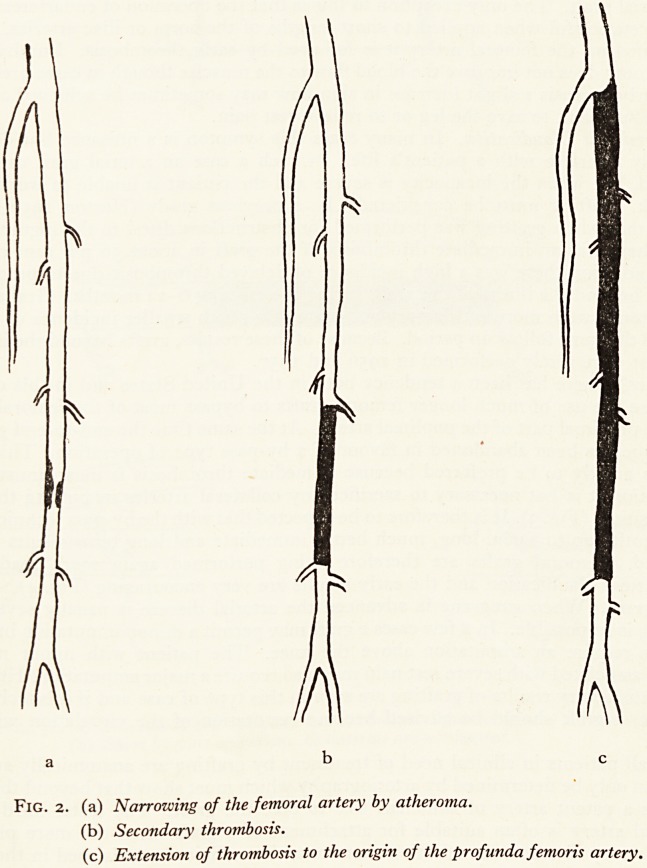


**Fig. 3. f4:**
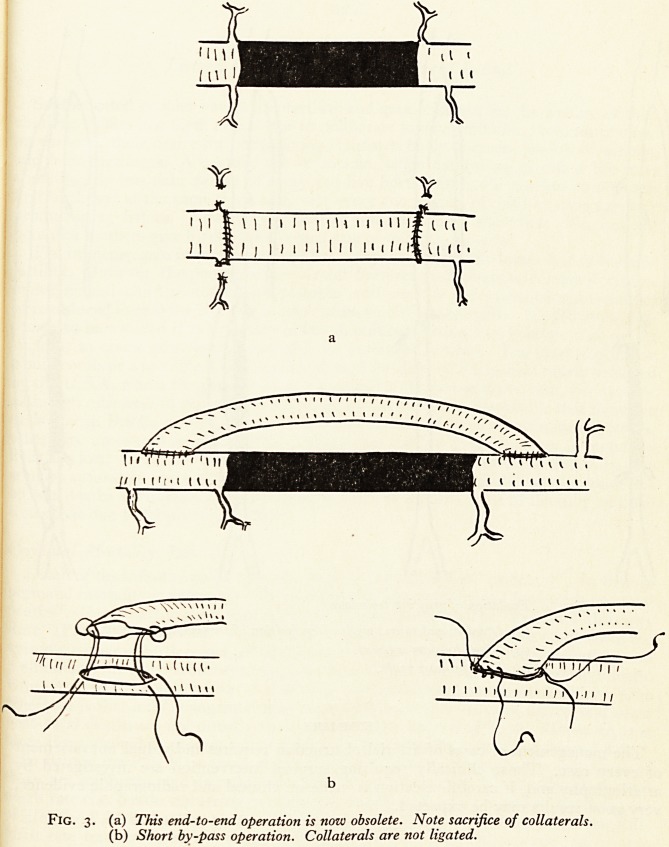


**Fig. 4. f5:**